# Surgical management of giant Brunner's gland hamartoma: case report and literature review

**DOI:** 10.1186/1477-7819-7-68

**Published:** 2009-09-02

**Authors:** Zoe A Stewart, Ralph H Hruban, Elliot F Fishman, Christopher L Wolfgang

**Affiliations:** 1 Department of Surgery, The Sol Goldman Pancreatic Cancer Research Center, Johns Hopkins Hospital, Baltimore, Maryland, USA; 2 Department of Pathology, The Sol Goldman Pancreatic Cancer Research Center, Johns Hopkins Hospital, Baltimore, Maryland, USA; 3 Department of Radiology, The Sol Goldman Pancreatic Cancer Research Center, Johns Hopkins Hospital, Baltimore, Maryland, USA

## Abstract

Brunner's gland hamartomas (BGH) are uncommon benign tumors of the duodenum forming mature Brunner's glands. We report here an unusual case of a giant BGH that was not amenable to endoscopic or surgical local resection thus requiring a pancreaticoduodenectomy for extirpation. The relevant literature is discussed.

## Background

Brunner's gland hamartomas (BGH) are uncommon benign tumors of the duodenum forming mature Brunner's glands. BGH have an estimated incidence of < 0.01% based upon review of one large autopsy series [1], and fewer than 200 cases have been reported in the English literature. These rare tumors have a low propensity for malignant transformation but can be confused with lesions of more oncological importance such as dysplastic duodenal adenomas or duodenal adenocarcinomas. Essentially all BGH can be managed endocscopically while duodenal adenocarcinoma requires more aggressive intervention. Thus recognition of BGH and differentiation from malignant tumors is critical for appropriate treatment. We report here an unusual case of a giant BGH that was not amenable to endoscopic or surgical local resection thus requiring a pancreaticoduodenectomy for extirpation.

## Case presentation

A 62-year-old Asian male presented to an outside institution with chief complaints of epigastric abdominal pain and reflux symptoms. Review of systems, past medical history, physical exam, and laboratory values were unremarkable. Family history was notable for pancreatic cancer in his father at the age of 92 years. An upper gastrointestinal contrast study was obtained and revealed a 6 cm mass within the duodenum that resulted in significant compromise of the lumen. A computed tomography (CT) scan demonstrated a cystic and solid lesion located within the duodenum and impinging on the head of the pancreas. Esophagoduodensocopy (EGD) and endoscopic ultrasound (EUS) demonstrated a submucosal, cystic lesion in the wall of the duodenum distal to the ampulla of Vater. The patient underwent an endoscopic ultrasound with multiple biopsies and fluid aspirations. Microscopic evaluation revealed benign glandular cells with reactive changes. No malignant cells were identified. Endoscopic un-roofing of the cystic lesion was performed. Clear viscous fluid was noted to emanate from the lesion and again pathology demonstrated only benign glandular cells with reactive changes. Despite this procedure the mass was noted to recur and grow in size over the next three 3 years. Over this time the patient did not experience vomiting or weight loss but did have significant worsening of his reflux symptoms.

**Figure 1:**
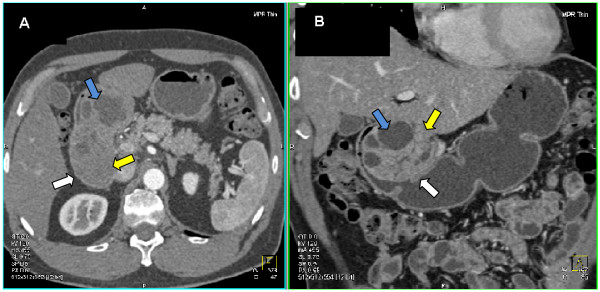
**Appearance of a Giant Brunner's Gland Hamartoma on Computed Tomography Scan**. (a) Contrast enhanced axial image in arterial phase of acquisition demonstrates an approximately 10 cm mass in duodenum extending into the gastric antrum (white arrow) with both solid (yellow arrow) and cystic components (blue arrow) (b) coronal display shows the intraluminal nature of the mass (white arrow) and its extent as well as again defining both the solid (yellow arrow) and cystic components (blue arrow).

**Figure 2:**
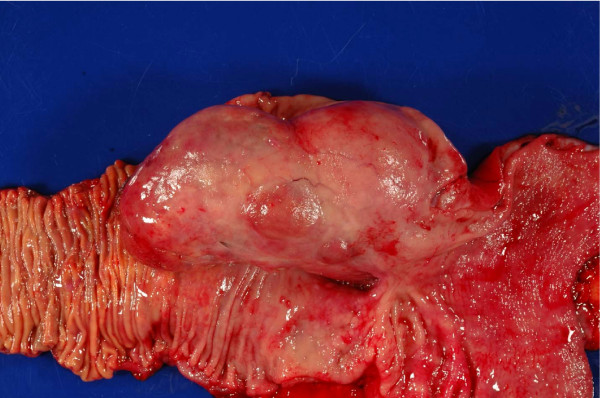
**Pancreaticoduodenectomy Specimen with a Giant Brunner's Gland Hamartoma**.

The patient was referred to our institution and evaluated by a multidisciplinary gastrointestinal oncology team. CT imaging at that time demonstrated a massive intraluminal mass extending from the antrum through the duodenum (Figure 1). Based on this finding and previous failed attempts at endoscopic management it was decided that this tumor could not be resected endoscopically. He was offered surgical exploration and resection. Preoperatively, it was felt that this lesion could be removed through a trans-duodenal local resection. At operation the tumor was found to have a broad-based attachment to the duodenal wall and a local excision was not possible (Figure 2). The patient underwent a pancreaticoduodenectomy. Surgical reconstruction was performed with a Peng end-to-end binding pancreaticojejunostomy as previously described [2] with the exception of placement of a 3.5 French plastic pediatric feeding tube as a pancreatic stent [3]. Three 10-mm Jackson-Pratt silicone drains were left at the pancreaticojejunostomy and hepaticojejunostomy anastomoses as previously described [3]. The patient advanced to a regular diet by postoperative day (POD) 6 but had amylase-rich drain output of less than 200 milliliters per day. As a result of the high-output postoperative pancreatic fistula, the patient was maintained on a low-fat diet and discharged home POD 19 with the drain that was removed in clinic POD 34.

**Figure 3:**
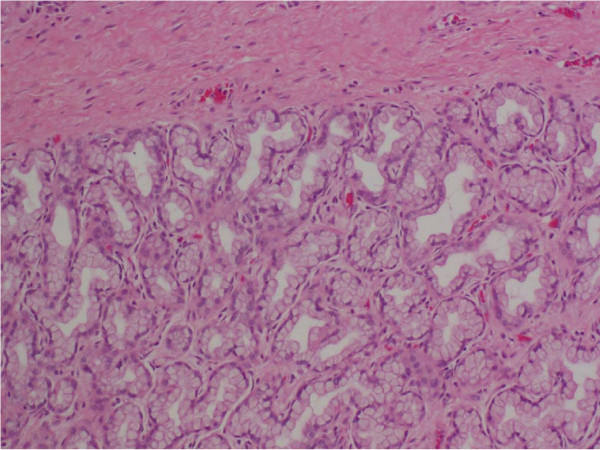
**Histopathological Appearance of a Giant Brunner's Gland Hamartoma stained with Hematoxylin and Eosin and viewed at 10×**.

Pathologic examination demonstrated a Brunner's gland hamartoma measuring 10.5 cm (Figure 3). No dysplasia or malignancy was seen within the entirety of the specimen. The lesion was composed of back to back mature Brunner's glands.

## Discussion

Brunner's glands are alkaline-secreting glands located in the submucosal layer of the duodenum. The majority of Brunner's glands are located in the first portion of the duodenum, with decreasing prevalence in the second and third portions of the duodenum. BGH follow this distribution, as a series of 27 patients with BGH found 70% in the duodenal bulb, 26% in the second portion of the duodenum, and 4% in the third portion [4]. As BGH grow they typically form polypoid, pedunculated masses. BGH has equivalent gender and race distribution with the age of presentation typically in the fifth or sixth decade of life. BGH is often an incidental finding during EGD or imaging studies as the majority of patients are asymptomatic. In symptomatic patients, clinical manifestations can include gastrointestinal bleeding, duodenal obstruction, abdominal pain, ampullary obstruction, or intussusception [4,5]. Chronic low-grade hemorrhage may lead to iron deficiency anemia [6]. At least one instance of a BGH causing pancreatitis, presumably due to obstruction of the Ampula of Vater, has been reported [7].

Differential diagnosis includes duplication cyst, leiomyoma, leiomyosarcoma, adenoma or adenocarcinoma, lymphoma, carcinoid tumors, heterotopic pancreatic or gastric tissue, or gastrointestinal stromal tumors. Pathologic review demonstrates the admixture of normal tissues including Brunner's glands, ducts, adipose tissue, and lymphoid tissue [4]. The cells have uniform nuclei and significant dysplasia is not seen. The pathogenesis of BGH remains unclear. It has been hypothesized that BGH is related to hyperacidity with compensatory growth of the alkaline-secreting Brunner's glands[5] or to *Helicobacter pylori *infection [8]. However, given the low incidence of these tumors, definitive studies into the etiology are lacking.

The description of BGH on computed tomography in the literature, particularly in the era of multi-slice helical CT scanners has been limited but varies from homogenous enhancement with intravenous contrast administration to heterogenous lesions with solid and cystic components [9]. EUS examination clearly demonstrates the submucosal origin of BGH and typically demonstrates heterogenous lesions with solid and cystic components [10].

There have been rare reports of malignant transformation of BGH in the literature. Brookes *et al *recently described a patient who presented with gastrointestinal bleeding secondary to a 2 cm BGH [11]. The lesion was removed endoscopically and final pathology revealed BGH with multiple foci of dysplasia [11]. These reports suggest that caution must be used when deciding on treatment algorithms for patients with presumed BGH as the potential for malignant transformation cannot be excluded.

Biopsies are typically indeterminate given the submucosal location of the lesions. Treatment options can include endoscopic removal for those lesions on a pedunculated stalk [5,8] to surgical resection for giant broad-based lesions [12]. The benign nature of BGH, and in most cases the lack of significant symptoms, makes endoscopic management of these patients the preferred initial mode of therapy. However, if endoscopic interventions fail surgical resection may be necessary in symptomatic patients or those in whom a malignancy is suspected. In the current case report, our patient was experiencing significant reflux symptoms and an attempt and endoscopic un-roofing was unsuccessful. We therefore planned to perform a trans-duodenal polypectomy. At operation, we found the lesion to have a broad-based stalk not amenable to this plan and a location that did not allow a duodenal sleeve resection. Moreover, the massive size and ulcerated appearance raised our level of concern of occult malignant degeneration. We therefore proceeded to perform a pancreaticoduodenectomy. This would seem to be a very unusual circumstance. Indeed, only two cases of a resection of BGH by pancreaticoduodenectomy have been reported in the literature [13,14]. In both cases the authors were concerned with malignancy. Similar to our case malignancy was not identified on final pathology.

## Competing interests

The authors declare that they have no competing interests.

## Authors' contributions

ZAS contributed to the study design, manuscript preparation and editing. RHH contributed to the evaluation of the histopathology, manuscript preparation and editing. EKF contributed to the interpretation of the cross-sectional images, manuscript preparation and editing. CLW contributed to the study design, manuscript preparation and final editing. All authors read and approved the final manuscript.

## Consent

Written informed consent was obtained from the patient for publication of this case report and accompanying images. A copy of the written consent is available for review by the Editor-in-Chief of this journal.
